# The Involvement of Endothelin-1 in Sepsis and Organ Dysfunction—A Novel Biomarker in Patient Assessment

**DOI:** 10.3390/biomedicines13102480

**Published:** 2025-10-11

**Authors:** Cristian Sorin Prepeliuc, Maria Antoanela Pasăre, Maria Gabriela Grigoriu, Ionela Larisa Miftode, Radu Ștefan Miftode, Andrei Vâță, Irina Iuliana Costache-Enache, Egidia Gabriela Miftode

**Affiliations:** 1 Doctoral School, “Grigore T. Popa” University of Medicine and Pharmacy, 700115 Iasi, Romania; 2 County Emergency Hospital “Mavromati”, 710221 Botoșani, Romania; 3 “Sf. Parascheva” Clinical Hospital of Infectious Diseases, 700116 Iasi, Romaniaemiftode@yahoo.co.uk (E.G.M.); 4 Department of Infectious Diseases, Faculty of Medicine, “Grigore T. Popa” University of Medicine and Pharmacy, 700115 Iasi, Romania; 5 Department of Internal Medicine I (Cardiology), Faculty of Medicine, “Grigore T. Popa” University of Medicine and Pharmacy, 700115 Iasi, Romaniaii.costache@yahoo.com (I.I.C.-E.); 6 Department of Cardiology, Clinical Emergency Hospital “Sf. Spiridon”, 700111 Iasi, Romania

**Keywords:** sepsis, endothelin-1, biomarker, endothelin receptor, multi-organ dysfunction, cardiovascular dysfunction, renal dysfunction, liver dysfunction, pulmonary dysfunction, neurological diseases

## Abstract

Sepsis represents a life-threatening organ dysfunction caused by a dysregulated host response to infection, and is considered a medical emergency. Therefore, quick diagnosis and treatment are required in order to improve survivability. Currently, patient evaluation in sepsis is based on the Sequential Organ Failure Assessment (SOFA) score to determine the severity of the disease; however, novel biomarkers are also actively researched with the aim to develop quicker diagnostic tools and better therapy. Endothelin-1 is one of the most potent vasoconstrictors found in the human body and is involved in the pathophysiology of both sepsis and other conditions involving organs that make up the SOFA score. In this narrative review, we aimed to gather information of this peptide’s multiple effects and to help determine whether or not it could prove a valuable biomarker in the evaluation of patients with multi-organ dysfunction in sepsis.

## 1. Introduction

The present definition of sepsis, established in 2016 by a task force convened by the Society of Critical Care Medicine and the European Society of Intensive Care Medicine, states that it represents a life-threatening organ dysfunction caused by a dysregulated host response to infection. In other words, it can be seen as a systemic response to an infection [1]. Therefore, in order to establish the diagnosis of sepsis, two criteria are required: a primary infection and the dysfunction of at least one organ, away from the main infection site.

According to the World Health Organization (WHO), five of the top ten mortality causes meet the criteria for sepsis; despite recent therapeutic advancements, it remains the most frequent cause of death amongst critically ill patients worldwide [2].

This is why sepsis and septic shock are considered medical emergencies. Present guidelines recommend that the disease should be treated and (if needed) resuscitated immediately, with the best results being achieved when performed within the first 3 h. The main risk factor for death in septic patients is heart failure, which is a frequent complication of sepsis. Once heart failure occurs, the mortality rate increases. Therefore, it is important to identify this complication as soon as possible. To that end, echocardiography should be performed in order to further assess the hemodynamic status of the patient. Myocardial insufficiency caused by sepsis is characterized by a decrease in left ventricular diastolic function and left ventricular ejection fraction (LVEF). However, at present, there are no clear diagnostic criteria for sepsis complicated with heart failure, and the limitations of ultrasonography have been extensively described in the literature [1,2].

Given this fact, new biomarkers implicated in the pathophysiology of sepsis need to be found in order to develop novel means of diagnosis and treatment. To that end, one such molecule could be endothelin-1.

First discovered in 1988 by Yanagisawa et al. [3] from the supernatant of porcine aortic endothelial cells, endothelin-1 (ET-1) is a potent vasoconstrictor and mitogen peptide, composed of 21 amino acids [2,3,4]. It was soon followed by the discovery of two other related peptides (endothelin-2 and endothelin-3), as well as their receptors and their activating enzymes (endothelin converting enzymes 1 and 2) [5]. All of these three peptides are the product of different genes, located on chromosomes 6, 1, and 20, respectively. However, ET-1 presents the highest concentrations in the human body out of the three molecules, and it is also the one best described in medical literature [2].

Although initially identified in endothelial cells, the production of ET-1 has now been reported in many cell types, such as cardiomyocytes, neurons, renal medulla, immune cells such as macrophages, dendritic cells, leukocytes, as well as fibroblasts. Furthermore, ET-1 has been found to be expressed basally in pulmonary epithelial cells and keratinocytes [2,5].

Although ET-1’s main effect is vasoconstriction, it also causes vascular cells fibrosis and stimulates production of reactive oxygen species. Furthermore, it activates transcription factors such as NF-κB, leading to the production of superoxide anion and cytokine secretion (including TNF-α, IL-1, and IL-6) and development of inflammation. Likewise, these transcription factors and proinflammatory cytokines can stimulate ET-1 production. However, ET-1 gene expression in endothelial cells can also be activated by some physical and chemical stimulants, through the binding of transcription factors such as activator protein-1, GATA-2, Smad, hypoxia inducible factor-1, and NF-κB [2].

In sepsis, the involvement of endothelin-1 has been observed in some studies [1,2,4], as detailed below (see point 4. Involvement in sepsis). As such, elevated values of serum ET-1 have been observed in septic shock, when it leads to the development of inflammation inside vascular walls (where it increases the expression of adhesion molecules on vascular endothelial cells and stimulates the aggregation of polymorphonuclear neutrophils), by increasing vascular permeability, cytokine release and leukocyte migration. This leads to the impairment of the vascular tone during sepsis, in part due to the abnormal production of cytokines such as nitric oxide (NO), prostacyclin, and endothelin [2].

Usually, endothelial cells in the endocardium affect the contractility of myocardial cells using multiple substances, such as ET-1, angiotensin II and nitric oxide, for signaling. The most frequently observed effects determined by the endocardial endothelium are heart failure, atrial fibrillation, and ischemia/reperfusion injury. If sepsis is complicated with heart failure, the patients may present with a systemic endothelial dysfunction, involving the endocardium and the vascular endothelium, which ultimately leads to an increase in hemodynamic load of the left atrium and an increase in synthesis and release of ET-1 and angiotensin II [1].

## 2. Materials and Methods

With this narrative review, our aim is to bring together the multiple effects of endothelin-1 described in the literature and determine whether or not this molecule could be a valuable biomarker in the evaluation of patients with sepsis and multi-organ dysfunction (specifically, organs that make up the SOFA score, used for sepsis evaluation). In order to achieve this goal, we searched the PubMed database for articles regarding this subject, published between 2020 and 2025, by using the keywords “Endothelin-1” and “Sepsis”, as well as “Endothelin-1” and “Cardiovascular disease”, “Kidney dysfunction”, “Liver dysfunction”, “Pulmonary dysfunction”, and “Neurological disease”. Based on these keywords, our search yielded 899 studies.

After our initial search, we decided to include in our analysis those studies that directly tackle the topic of which frequent pathophysiological processes endothelin-1 directly plays a role in. Furthermore, we analyzed papers describing situations and/or diseases where this peptide serves as a diagnostic or prognostic biomarker, as well as a treatment monitoring biomarker. In order to provide a broader view of ET-1, we decided to include in our summary all types of studies focusing on these subjects.

We excluded those articles that did not focus specifically on the effects of endothelin-1, but rather on other subjects and/or other biomarkers. We also excluded studies describing ET-1’s roles in physiological processes.

In the end, after excluding papers deemed irrelevant for the subject, we chose 57 articles for the final analysis and inclusion in this review. For a more facile approach to the review, we also grouped them into six categories, depending on their focus on sepsis or on the organ dysfunction used for determining the SOFA score.

## 3. Main Effects of Endothelin-1

As mntioned beforehand, the endothelins are peptides consisting of 21 amino acids, three isoforms being identified in the human body: endothelin-1 (ET-1), endothelin-2 (ET-2), and endothelin-3 (ET-3). All three are synthesized by different genes, which are located on chromosomes 6, 1, and 20, respectively—EDN1, EDN2, and EDN3. However, endothelin-1 is the most abundant in our organism and is also the isoform best described in the literature. The transcription of the gene responsible for the production of endothelin-1—EDN1—is controlled by multiple factors, such as hypoxia inducible factor 1 and activator protein 1, which enables the regulation of ET-1 secretion and release by different cells and tissues in different physiological or pathological conditions [2,3,4,5,6].

**Figure 1:**
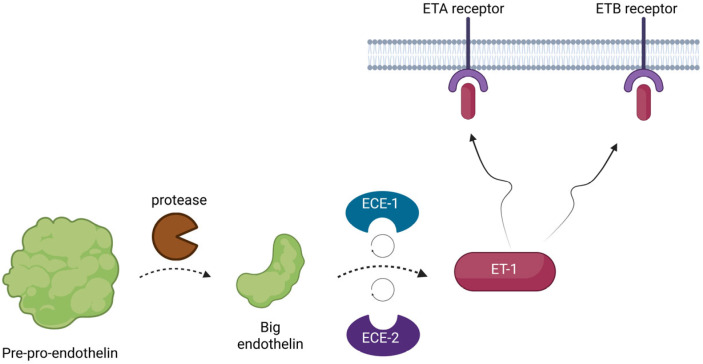
Synthesis and receptors of Endothelin-1.

The three genes initially encode a larger peptide, known as pre-pro-endothelin. This precursor is biologically inert, but it is quickly activated by proteases. At first, the pre-pro-endothelin is cleaved by the enzymes into another smaller peptide, known as big-endothelin, another inactive precursor. Afterwards, this precursor is once more converted by two membrane-bound metalloproteases, known as endothelin-converting enzymes 1 and 2 (ECE1 and ECE2), into its final form and it is released into the interstitial space and in smaller concentrations into the circulation [4,5,6] (see Figure 1).

Although it is mainly produced by endothelial cells (hence its name), ET-1 is in fact synthesized by other cells as well, such as leukocytes, macrophages, fibroblasts, keratinocytes, cardiomyocytes, neurons, pulmonary epithelial cells, and also by the renal medulla. Many factors are involved in its secretion, and studies show that the expression of endothelin components is regulated by the circadian rhythm, cell stress, and epigenetics [2,4,6]. High values of serum ET-1 have been observed in acute and chronic stress, hyperosmolality, high sodium intake, hypoxia, obesity, inflammation, insulin resistance and type 2 diabetes, cardiovascular diseases, asthma, as well as infectious diseases [2,6]. For example, ET-1 is synthesized and released by the endothelial cells when stimulated by epinephrine, thromboxane, vasopressin, angiotensin, insulin, and cytokines. Out of all three endothelins, the transcriptional regulation of ET-1 is the one best described in the literature. Currently, researchers have identified ten signaling pathways involved in the expression of ET-1 [2,4,5,7].

After ET-1 is synthesized, research indicates that it can be stored inside the endothelial cells in cytoplasmic secretory vesicles, but it is unknown exactly which organelles are responsible for its storage. Most studies suggest that endothelin-1 is kept primarily within Weibel-Palade bodies in the endothelial cells (together with pro-ET-1 and ECE-1, suggesting that they could be the site of ET-1 synthesis/conversion); however, there are clues that it can also be found inside vesicles formed from the plasma membrane [4].

The main effect exerted by ET-1 is considered vasoconstriction (both directly or indirectly, by stimulating the growth of vascular smooth muscle cells), thus influencing blood pressure and the basal vascular tone. However, other identified secondary effects are fibrosis of the vascular cells, increased production of mitochondrial reactive oxygen species, transient Ca^2+^ increase, and enhanced consumption of ATP. It also mediates the glycosaminoglycan (GAG) chain hyperelongation on proteoglycans. Furthermore, as previously mentioned, endothelin-1 can activate transcription factors such as NF-κB, which leads to the production of superoxide anion and cytokine secretion (including TNF-α, IL-1, and IL-6) and development of inflammation. Likewise, these transcription factors and proinflammatory cytokines can stimulate the production of ET-1. However, ET-1 gene expression in endothelial cells can also be activated by some physical and chemical stimulants, through the binding of transcription factors such as activator protein-1, GATA-2, Smad, hypoxia inducible factor-1 and NF-κB. Given these effects, other studies have described the involvement of endothelin-1 in the development of atherosclerosis [2,4,8].

ET-1 exerts its effects by binding to two types of receptors—ETA and ETB. These are part of the transmembrane Class A G protein-coupled receptor family (GPCR), which involve heterotrimeric G proteins for signaling and are found in a variety of tissues (endothelium, vascular smooth muscle cells, adipocytes, hepatocytes, etc.). The G proteins are composed of a Gα subunit (which, in turn, contains four families—Gq/11, Gi/o, Gs and G12/13—that activate or inhibit kinase pathways, gene expression or the production of secondary messengers) and an obligate Gβ/Gγ heterodimer. Given the numerous G protein pathways that ET-1 can activate, we might be able to explain the multitude of roles ET-1 plays inside our body, such as ion transport, vascular permeability and inflammation, besides blood flow and muscle contraction control [2,4,5,6,8]. Furthermore, given the presence of endothelin-1 receptors in insulin-sensitive tissues, it might suggest its involvement in the pathogenesis of insulin resistance [6].

ETA receptors are mostly located in the vascular smooth muscle tissue, where they are involved in vasoconstriction, cellular proliferation, the generation of reactive oxygen species, and inflammation (playing a part in the proinflammatory and pro-atherogenic effect). However, studies show that the ETA receptors located in the brain might play a role in reducing the mortality of animals with sepsis [2,5,8]. Also, some studies show that in rat fibroblasts, the activation of ETA receptors leads to their differentiation into adipocytes and lipolysis stimulation [6]. They are more selective than ETB receptors, demonstrating a high affinity for ET-1, moderate affinity for ET-2, and little affinity for ET-3, respectively [5].

**Figure 2:**
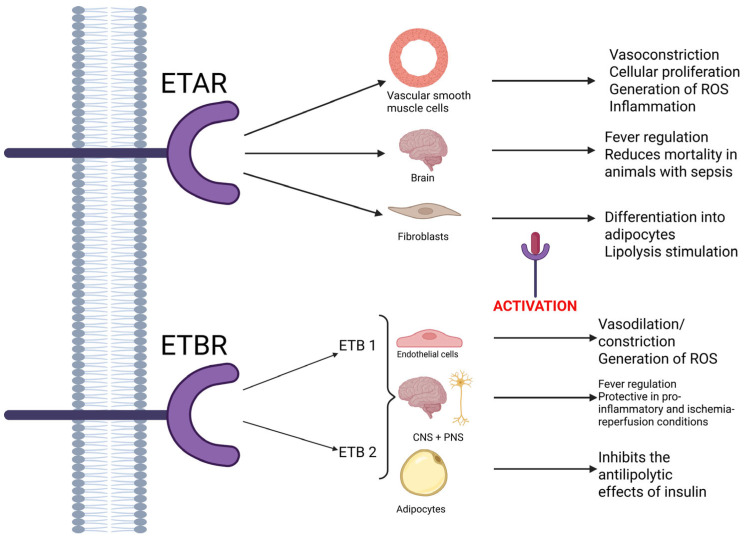
Effects of endothelin receptors.

In contrast, ETB receptors are nonselective and bind to all isoforms of endothelin with equal affinity [5]. Two subtypes of receptors have been identified: ETB1, which are expressed on endothelial cells and play a part in vasodilation mediated by nitric oxide, and ETB2, which are predominantly involved in contraction. Both these subtypes, along with ETA receptors, are involved in the generation of reactive oxygen species. In the central nervous system, ETB receptors are also known to be involved in the appearance of fever, while stimulation of ETB1 receptors provides tissue protection in proinflammatory and ischemia–reperfusion conditions in the peripheral and central nervous system. Furthermore, some authors have pointed out the fact that ETB receptors could be connected to the clearance of ET-1 [2,4,5]. Also, in adipocytes, ETB receptors seem to inhibit the antilipolytic effects of insulin, once activated. As such, the effects of ET-1 depend on the type of activated receptor [6]. A schematic of all these effects is available in Figure 2, listed below.

Other than the G proteins, evidence shows that these receptors could also be coupled to β-arrestin, which may play a part in the downregulation of G protein signaling via the internalization of ET receptors for recycling or degradation. In order to sustain this theory, there is also evidence that ET-1 could intensify signaling via the β-arrestin pathway [4].

Although ET-1 mainly plays its role in vasoconstriction and dilation, endothelin signaling is also involved in the development of tissues derived from neural crest cells (such as the craniofacial bones, cardiac outflow tract, and the enteric nervous system), one of the most important steps in vertebrate evolution. This type of signaling can promote neural crest cells to migrate, proliferate, differentiate, or maintain their state, depending on the involved ligands, receptors, cells, and tissue types. Furthermore, ET-1—ETA signaling was proven to be essential in certain stages of cardiac development, such as remodeling of the cardiac outflow tract, ventricle septation and valve formation, as well as the cardiac conduction system [5].

Besides vasodilation and constriction, ET-1 is considered an atherogenic peptide because it stimulates migration and proliferation of smooth muscle tissue within the vascular wall, helps attract circulating monocytes through chemotaxis, activates macrophages, participates in the formation of fibrous tissue, inhibits the production of nitric oxide and leads to inflammation in the vascular wall. In fact, in patients with chronic kidney disease, guidelines suggest that ET-1 tissue immunoreactivity is the main predicting factor of progression of atherosclerosis [9].

The pathways through which this peptide leads to atherosclerosis are not yet fully understood and are still being researched at the present time. For example, in a study performed by Brewster et al., the authors found out that, besides its direct effects on cellular markers, endothelial inflammation markers and nitric oxide (NO) production, endothelin-1 also induces the production of endothelial microvesicles. When these vesicles are derived from ET-1, they increase inflammation (mainly by increasing total and activated NF-κB transcription factor, which leads to the release of IL-6 and IL-8) and reduce NO production by the endothelial cells (through the downregulation of endothelial nitric oxide synthase), in addition to exerting a similar effect to ET-1, amplifying the pro-atherogenic effect. High concentrations of endothelial microvesicles were also identified in other afflictions associated with elevated values of ET-1, such as hypertension, acute coronary syndromes, heart failure, and ischemic stroke. However, the mechanism through which ET-1 induces the production of endothelial microvesicles remains unknown [9].

In another study performed by Wang et al. [7], the authors analyzed ET-1’s effect on the function of cardiomyocytes, cardiovascular function, and its role in chronic coronary syndrome. Given its involvement in endothelial dysfunction, inflammation, and atherosclerosis, the authors indicate that ET-1 also plays a role in the development and progression of coronary artery disease and heart failure following myocardial infarction. Their research shows that this peptide can be considered an important biomarker for cardiovascular risk classification and a prognostic marker for long-term cardiovascular and all-cause mortality, as well as secondary effects [7].

Other studies have shown that endothelin-1 has many more effects, such as mediating the transactivation of protein tyrosine kinase receptors (alongside thrombin, angiotensin II, and other GPCR agonists) and stimulating the synthesis and release of proteoglycans with longer GAG chains [8]. Furthermore, in an article published by Babaahmadi-Rezaei et al. [8], the authors show that vascular smooth muscle cells treated with ET-1 present increased time-dependent levels of Smad2 linker region phosphorylation via a pathway involving NOX and p38 MAP kinase, as well as high mRNA expression of C4ST-1 and ChSy-1 (because the expression of these genes involves ET receptor-mediated transactivation of TβR1) [8].

Certain effects of ET-1 have also been observed in the gastrointestinal tract, where it plays a part in the control of ion transport, gut absorption and secretion, and peristalsis (by inducing contraction of the smooth muscle cells) [4].

Another pathophysiological mechanism in which ET-1 is involved is the immune response. As mentioned before, endothelin-1 indirectly leads to the transcription and release of inflammatory cytokines (such as tumor necrosis factor-α (TNF-α), interleukin-1 (IL-1), and interleukin-6 (IL-6), from monocytes) and also promotes chemotaxis along with these cytokines. Moreover, these proinflammatory markers also stimulate the synthesis and release of ET-1, leading to positive feedback. Other ET-1-dependant mechanisms related to the immune response include the regulation of vascular permeability and the increase in hematocrit (in studies on rats administered with ET-1) [4].

## 4. Involvement in Sepsis

Sepsis and septic shock are considered medical emergencies. The current definition of sepsis, Sepsis 3.0, states that it represents a life-threatening organ dysfunction caused by a dysregulated host response to infection [1,2,10,11]. Guidelines suggest that, once diagnosed, the disease should be treated and resuscitated immediately, including initial resuscitation within the first 3 h [1].

According to epidemiological research, sepsis is the most frequently identified cause of end-stage organ failure, following reduced tissue perfusion and hypoxia. In 2020, approximately 49 million cases were reported worldwide, with 11 million deaths, representing 20% of all global deaths [11]. Furthermore, in recent years, reports show a significant increase in sepsis-induced mortality rates, especially in intensive care units [10].

Endothelin-1 is proven to play an important role in the pathophysiology of sepsis and its complications; elevated plasma levels of ET-1 have been identified in hypotensive septic animals [2]. One of these complications is septic shock. This is defined as a hyperdynamic state with increased cardiac output and reduced peripheral vascular resistance, resulting in heart failure and ultimately in multiple organ failure. During septic shock, multiple vasoactive substances are released from the endothelium, including endothelin-1 [10,11]. In addition, sepsis can determine cardiac dysfunction through reduced cardiac contractility due to volume depletion, high vascular permeability, and low vascular tone. Other than the cardiac involvement, during sepsis, acute kidney injury can be identified; the available data revealed that approximately 40% of patients develop this complication [11].

Numerous studies have described the involvement of ET-1 in the pathophysiology of sepsis [1,2,4,10,11,12,13,14,15,16,17]. A summary of the findings from these studies can be found in Table 1.

Physiologically, endothelial cells play an important role in infections, in order to limit the dissemination of the systemic response, control leukocyte recruitment, and facilitate bacterial elimination. In sepsis, the regulation of endothelins (especially ET-1) is one of the first systems affected, leading to modifications in blood flow (through systemic vascular dilation, which does not respond to pressor medication), perfusion, and capillary permeability. High levels of this peptide have been identified in acute heart, kidney, lung, and liver injury (especially during sepsis), as well as septic shock, promoting inflammation inside vascular walls through increased vascular permeability, cytokine release, and leukocyte migration. Therefore, during sepsis, an impairment of the vascular tone due to the dysregulated production of vascular permeability, cytokine release, and leukocyte migration can be observed. Also, these massive inflammatory and oxidative stress responses, combined with endothelial damage, microvascular dysfunction, and hypoxemia, lead to sepsis-induced organ failure [2,10,11].

Therefore, during sepsis, elevated levels of ET-1 can be identified, leading to endothelial dysfunction (which, in turn, contributes to the worsening of other preexisting conditions, such as vascular complications in diabetes or cardiovascular diseases) [12], but studies show that this also determines renal vasoconstriction and hypoperfusion, leading to dysregulated electrolytes and metabolic acidosis. Research performed by Al-kadi et al. [11] on rats in which sepsis was induced by cecal ligation and puncture shows, among others, that by activating ETB receptors, Na reabsorption in the collecting ducts is inhibited and R-type calcium channels and Na+/Ca^2+^ exchangers are open. This can result in increased levels of intranuclear and cytoplasmic Ca^2+^. Furthermore, other studies have shown that the inhibition of ETA receptors can correct the abnormal regulation of Ca^2+^ [11].

In the same study, the authors measured ET-1 serum levels in the septic rats and observed increased values of this peptide. They suggest that this might be the consequence of increased production of ET-1 and decreased pulmonary and renal elimination, the marker playing an important role in vascular and organ dysfunctions during sepsis and septic shock [11].

**Figure 3:**
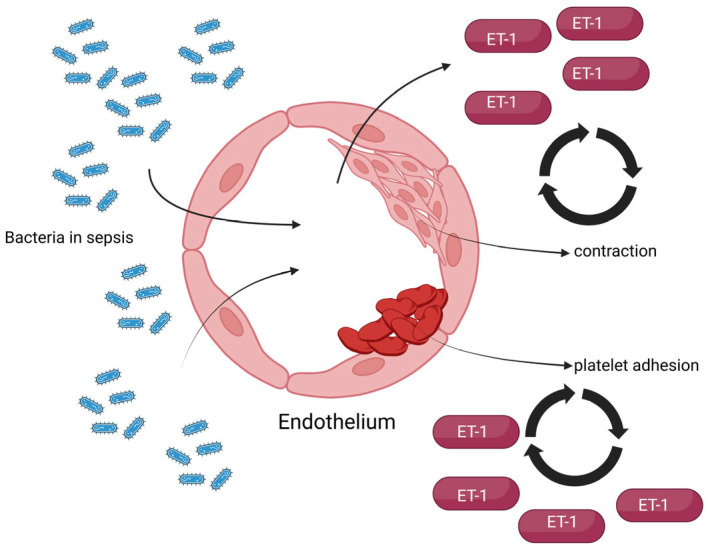
ET-1 effects on the endothelium during sepsis.

On the other hand, regarding the endothelium of septic patients, the endothelial basement membrane is exposed following damage, which favors platelet adhesion and aggregation and leads to the initiation of the coagulation cascade. Given the vasoconstrictive effects of ET-1, an elevation in its serum concentration can lead to hypercoagulation, endothelial ischemia, and hypoxia, which in turn further increases ET-1 concentration [13]. A simple representation of these pathophysiological effects is presented in Figure 3.

Further research suggests that endothelin-1 might promote edema formation, especially in septic patients. This occurs due to the high levels of heparin-binding protein, a protein that increases vascular permeability and contributes to edema formation during endotoxemia [2].

In another study performed by Lomba et al. [12] on lab rats, the authors showed that ET-1 is also involved in fever induced by bacterial endotoxins, such as lipopolysaccharide (LPS). In their experiments, they injected rats with LPS, and after 3 h, they identified an increased concentration of ET-1 in the cerebrospinal fluid and decreased levels of big-endothelin. Additionally, they proved that the fever induced by LPS can be blocked by administering ETB receptor antagonists, and by ETA receptor antagonists as well (but only if the medication is injected later, 2 h after administering LPS). These results would suggest that ET-1 is involved in the febrile response induced by lipopolysaccharide initially by activating ETB receptors and in later stages, through ETA receptors activation. Therefore, the involvement of this biomarker in fever induced by bacterial or fungal infections can be deduced [14].

Besides animal experiments, there is proof in the literature that direct administration of ET-1 in humans can determine sepsis-like cardiovascular changes (for example, decreased cardiac output, pulmonary artery vasoconstriction, or impairment of renal and splanchnic circulation), as well as dysfunction of the liver and lungs. In the heart, besides reducing the output, ET-1 mainly exerts its vasoconstrictive effect, determining an increase in vascular resistance and permeability in addition to increased fluid flux into the extravascular space. In the end, this effect results in the development of the hypodynamic state of septic shock [2].

Given all these roles that ET-1 plays during sepsis, high serum levels of this peptide are correlated with increased severity and mortality of septic patients [2,10,15]. Cohort studies revealed that patients with sepsis presented higher levels of ET-1 than healthy subjects, while patients with severe sepsis had significantly increased levels than those with mild forms of the disease. Furthermore, ET-1 concentrations seem to be higher in deceased patients in comparison to surviving ones [2]. It is worth mentioning that, besides the blood, elevated values of ET-1 have been identified in other tissues during sepsis, such as the kidneys, lungs, and liver [10].

In order to further confirm the involvement of endothelin-1 in sepsis, some researchers performed experiments on septic animals with different medications. For example, animals in septic shock injected with ET-1 antiserum presented reduced shock-induced damages [10]. In other cases, treatment with endothelin system antagonists ameliorated the inflammatory syndrome and led to improvement of normal functions in the cardiac, renal, pulmonary, or gastrointestinal systems, in addition to conferring protective effects in cancer and COVID-19 [11]. Additionally, a study by Şehitoğlu et al. [10] on rats divided into three groups (control, sepsis induced by cecal ligation, and perforation and treated with thymol) showed that both ET-1 gene expression (in lung, kidney, and liver tissue samples) and serum levels were significantly decreased in the thymol group and increased in the sepsis group [10].

In another study by Zhu et al. [1], ET-1 levels in patients with simple sepsis and sepsis complicated with heart failure were evaluated. In their research, it is worth noting that some patients had prior endocardial and vascular endothelial dysfunction, which influenced the ET-1 detection level. However, their final results showed that ET-1 was significantly increased in patients with heart failure, in comparison to those with simple sepsis. These values also positively correlated with levels of an inflammatory marker known as CXCL8, as well as with the Sequential Organ Failure Assessment (SOFA) and APACHE II scores, and negatively correlated with heart function parameters (for example, left ventricular ejection fraction). Therefore, these results suggest that endothelin-1 can indicate the severity of sepsis and can be considered an independent risk factor for sepsis complicated with heart failure [1].

Regarding sepsis in the pediatric population, a paper published by Xu et al. [13] revealed the involvement of ET-1 in children, who are more susceptible to infections that could develop into sepsis, septic shock, and multi-organ dysfunction. Patients with sepsis were divided into two groups based on their prognosis, and the authors measured their serum ET-1 levels. The results showed that in the poor prognosis group, with longer hospital stays and higher treatment costs, ET-1 values were significantly higher, correlating with the severity of sepsis. An interesting aspect of the study is represented by the fact that endothelin-1 values and sepsis severity negatively correlated with levels of plasmatic total cholesterol, suggesting that both these biomarkers could predict the outcome of septic children. One possible explanation for this result, which the authors discuss, could be the fact that in sepsis, endothelial cells are damaged, releasing ET-1 in the bloodstream, leading to an imbalance of vasoactive factors [13].

Another type of lesion associated with septic shock, which leads to an increase in ET-1 levels (through the activation of endothelial cells), is endothelial glycocalyx shedding. This type of damage is usually described following intravenous fluid administration as treatment for shock. Present guidelines recommend this type of treatment as the first line for the haemodynamic resuscitation of patients with septic shock; however, multiple studies suggest that intravenous fluids can cause endothelial glycocalyx shedding, possibly through hypervolaemia or haemodilution. In order to verify this theory, Macdonald et al. [16] performed a study on adult patients with septic shock, in which they measured the levels of different biomarkers involved in endothelial damage (including ET-1). The authors noted that, with correct treatment, the concentration of endothelin-1 gradually decreased over time. However, their results did not identify a correlation between the administered fluid volume and any of the studied biomarkers, although they did observe a significant correlation between endothelial glycocalyx shedding and ET-1 [16].

In septic patients, reactive hyperemia is often identified. This is why Malheiro et al. [17] researched whether this effect (measured by peripheral arterial tonometry) is somehow correlated with the severity of endothelial dysfunction by measuring biomarkers such as ET-1. It is worth noting that, contrary to previous evidence, their results showed no significant differences in ET-1 levels between the control group and the septic patients’ group, although the selected groups did not differ significantly regarding comorbidities, both groups having levels below the detection limit. As such, the authors suspect that ET-1 levels in patients with numerous comorbidities and critical illness may not vary, even when suffering from an infection, or that ET-1 plasma quantification may not be sensitive enough in this population [17].

In conclusion, given its many effects, especially its vasoregulatory role, ET-1 is involved in many processes of the pathophysiology of sepsis, as proven by studies in the literature. However, the exact mechanisms through which sepsis develops remain unknown and need further research.

## 5. Involvement in Cardiovascular Dysfunction

Given the fact that its primary effect is vasoconstriction, ET-1 can directly or indirectly determine a multitude of cardiovascular changes and is therefore implicated in numerous pathophysiological mechanisms underlying this system and conditions such as coronary atherosclerosis and disease [4,7], arterial hypertension [18], cardiac fibrosis [19] and heart failure [20,21,22].

As mentioned beforehand, in patients with sepsis and/or septic shock, at the cardiac level, this peptide can contribute to the decrease in cardiac output and the increase of:

(1) Vascular resistance and permeability;
(2) Flux of fluids into extravascular space (resulting in the hypodynamic state of septic shock);
(3) Activity of the sinoatrial node (resulting in tachycardia) [2].

Alongside nitric oxide (NO), prostaglandins, and angiotensin II, endothelin-1 can also act through paracrine signaling, inside the endocardial endothelium, and modify the contractility of myocardial myocytes. In patients with heart failure, atrial fibrillation, and ischemia/reperfusion injury, dysfunction of both the endocardium and the vascular endothelium can be observed, resulting in an increased load in the left atrium and increased synthesis of ET-1 and angiotensin II [1,2].

Furthermore, it has been reported that, through the downregulation of FKBP12.6 and SERCA2a (two cardiac enzymes involved in myocyte contraction and relaxation, which are related to the endothelin system), ET-1 also plays a part in sepsis-induced acute heart failure [2]. This correlation is important because myocardial dysfunction in septic patients is one of the most frequently observed complications, patients usually presenting acute onset and quick progress. The pathogenic mechanisms that eventually lead to this condition involve multiple factors, such as mitochondrial dysfunction, dysregulation of inflammatory mediators, oxidative stress, and endothelial dysfunction, among others [1,2]. Therefore, given the severity of this disease, we need to search for markers of sepsis and heart failure in order to improve the patients’ outcome.

The effects of ET-1 on the heart have been extensively studied. A summary of the studies regarding this subject that we analyzed for this review is listed in Table 2.

Reports have proven that in the heart, ET-1 is stored after synthesis inside the Weibel–Palade bodies of the myocytes (along with its precursors). Once released, it modulates coronary artery tone and also directly affects the muscle, reducing cardiac output. It has been reported that, in patients with atypical chest pain, after infusing the left coronary artery with an ETA receptor antagonist, left ventricular dP/dt was reduced, which might suggest that ET-1 has a positive inotropic role. To further demonstrate this effect, other studies have shown that the myocytes from the left ventricle are significantly more shortened when ET-1 is directly applied to them. Furthermore, this peptide proved to increase contractility in both the left ventricle and the right atrium as well. However, due to a high expression of ETB receptors in the atrium, this effect developed following an initial transient reduction in contraction. This result suggests that the positive inotropic effect of ET-1 is, in fact, mediated by ETA receptors [4].

### 5.1. Endothelin-1 in Arterial Hypertension

One of the most important pathologies in which endothelin-1 is involved is arterial hypertension, especially when resistant or difficult to control. The deletion of ET-1 or ETA receptor genes lead to minor elevations in blood pressure values [18]. There is also a theory which states that this biomarker might be increased before even before changes in blood pressure values are observed, and these values persist as the disease develops. Evidence to support this theory was reported in some studies on hypertensive rats, but the results from later studies showed no proof of elevated ET-1 levels in the early stages of hypertension. However, in later stages of the disease, high levels could be clearly observed across multiple studies. Moreover, available data suggest that ET-1 plays a more important role in malignant hypertension, serum levels being significantly higher in rats treated with deoxycorticosterone acetate salt, in order to mimic malignant hypertension, compared to controls [4]. Additionally, in this type of rat, as well as stroke-prone spontaneously hypertensive rats, the elevated levels of ET-1 prompt the hypertrophic remodeling of large and small arteries [18].

Other theories suggest that the vasoconstrictor effects of ET-1 manifest only under certain pathophysiological conditions, and only then does it determine a rise in blood pressure levels. However, this theory has not yet been proven through studies, given the fact that ET-1-dependent vasoconstriction can be observed in healthy humans as well, and blocking the ETA receptors lowers blood pressure values [18].

It is unclear, though, where ET-1 is stored in hypertensive patients and which mechanisms are used for its release during arterial hypertension. Experimental rat models with inducible, endothelium-specific ET-1 overexpression exhibit sustained ETA receptor-mediated hypertension, vascular remodeling, endothelial dysfunction, and renal injury. Blocking the ETA receptors mitigates aldosterone-induced cardiac and vascular fibrosis, implicating ET-1 in aldosterone’s pathogenic effects. In humans, essential hypertension is associated with heightened sympathetic responses to endogenous ET-1. Patients with resistant hypertension undergoing renal denervation show reduced sympathetic activity and plasma ET-1 levels. Additionally, in salt-sensitive hypertensive individuals with suppressed renin activity, salt depletion enhances catecholamine-stimulated ET-1 release [18].

Another potential mechanism was reported in patients with preeclampsia. By treating pregnant mice with IgG from women with preeclampsia, the expression of preproET-1 mRNA was increased, and by administering ET receptor blockers, hypertension was ameliorated. Additionally, by treating human placental villous explants, human umbilical vein endothelial cells (HUVEC), and immortalized trophoblasts with IgG in vitro, the secretion of ET-1 increased following the activation of angiotensin II type 1 receptor and an increase in TNF-α and IL-6 [4].

### 5.2. ET-1 in Coronary Atherosclerosis, Coronary Artery Diseases, and Ischaemia

As mentioned earlier, one of the most important conditions in which endothelin-1 is implicated is atherosclerosis. Numerous studies on experimental animal models and humans with atherosclerosis showed increased levels of ET-1 both in circulation and in tissues. The mechanisms underlying the pathophysiology are still being discussed to this date, but there is proof in medical literature for some processes where ET-1 is involved. For example, in patients with coronary atherosclerosis, acetylcholine administration induced vasoconstriction rather than vasodilation, indicating endothelial dysfunction and a pathological shift favoring ET-1 release over nitric oxide. Plasma ET-1 levels, already elevated, increased further post-administration. Furthermore, a significant correlation was observed between plasma ET-1 levels and the number of plaques in symptomatic atherosclerosis. Immunohistochemical analyses revealed high ET-1 expression in endothelial and smooth muscle cells, particularly in active lesions, as well as in macrophages, suggesting its role in plaque progression and immune cell recruitment [4].

Other than all these afflictions, ET-1 can also act as a predictor factor for coronary artery disease and other microvascular dysfunction, suggesting it could be directly involved in the development of these diseases. Both plasma levels of the precursor big endothelin-1 and tissue levels of ET-1 are correlated with the severity of coronary artery disease [4,9]. It also plays an important role in the development of chronic coronary syndrome. In the study performed by Wang et al. [7], which we previously mentioned, the authors measured ET-1 levels in two groups of patients with chronic coronary syndrome, based on the patients’ Gensini score (a score meant to assess the coronary artery lesions through coronary angiography, patients being divided into a high Gensini score group and a low Gensini score group). Their results showed that serum ET-1 levels positively correlate with the Gensini score and, by default, with chronic coronary syndrome progression and severity, while also negatively correlating with nitric oxide levels [7].

Emerging evidence also suggests a significant role of ET-1 in the pathophysiology of ischaemic heart conditions. Elevated plasma ET-1 levels have been positively correlated with the severity of coronary artery disease in patients with recent myocardial infarction or persistent chest pain, as well as with the likelihood of requiring revascularisation procedures such as coronary artery bypass grafting. However, the involvement of ET-1 in patients with angina, but without substantial coronary artery disease, remains to be clearly defined. Additionally, experimental animal models further indicate that cardiac ischaemia may stimulate ET-1 production within both macrovascular and microvascular compartments. In pigs subjected to prolonged ischaemia and subsequent reperfusion, plasma ET-1 levels remained elevated. It is worth noting that the ischaemic myocardium exhibited a marked increase in ET-1 mRNA expression, predominantly within cardiomyocytes, suggesting these cells are a primary source of ET-1 synthesis during reperfusion. Furthermore, during ischaemic events, ET-1 appears to exert direct cytotoxic effects on cardiomyocytes. In vitro studies using cultured neonatal rat myocytes have demonstrated that ET-1 induces a dose-dependent increase in lactate dehydrogenase release, indicative of cell damage, during simulated ischaemia. These effects were absent in normal conditions, and the potential protective impact of therapy with ET receptor antagonists remains unexplored [4].

### 5.3. ET-1 in Cardiac Fibrosis

Another example of a process in which endothelin-1 is involved is the pathogenesis of cardiac fibrosis [19]. In patients with heart failure and animal models of cardiac fibrosis, ET-1 can be found in elevated concentrations in the heart. ET-1 promotes fibrosis, vasoconstriction, proliferation, and adhesion via ET receptors, especially ETA. ETA receptor signaling is primarily profibrotic, mediating myofibroblast differentiation, collagen matrix contraction, and collagen deposition, particularly in hypertensive models. The mechanism involved in fibrosis starts from ET receptors signaling, which activates downstream pathways such as mitogen-activated protein kinases (MAPKs), particularly ERK1/2, which play a central role in ET-1-mediated fibrotic responses and myocardial hypertrophy. In vascular smooth muscle cells, ERK1/2—but not p38 MAPK—is essential for connective tissue growth factor production. ERK1/2 activation by ETA receptors occurs via a Gq/11-dependent, PLC-independent mechanism, though its role in human cardiac fibroblast fibrosis remains unclear. Inhibition of ET receptors, notably with bosentan, has been shown to attenuate cardiac fibrosis, improve cardiac remodeling, and enhance survival in experimental models and patients with heart failure [19]. Given the fact that ET receptors determine fibroblast activation and myofibroblast differentiation (resulting in a higher expression of α-smooth muscle actin and collagens, cardiac fibrosis and ultimately to heart failure) when stimulated by ET-1, in a study published by Duangrat et al. [19], the authors sought to analyze this effect, the steps leading to it and whether or not reversing myofibroblast differentiation could represent a potential therapeutic strategy. They performed this by incubating human fetal cardiac fibroblasts with different doses of ET-1, both without and with ET receptor antagonists pre-treatment. Their results showed that receptor stimulation and treatment with ET-1 lead to fibroblast proliferation, as well as increased α-SMA and collagen I synthesis in these cells, while only ETA receptor stimulation determines profibrotic effects, fibroblast activation, and myofibroblast transdifferentiation (by the ETA receptor/Gαq pathway). Furthermore, they proved that, as stated earlier, cardiac fibrosis is induced by ET-1 stimulation of ET-receptors through a mechanism involving ERK1/2 via the ETA receptor/Gαq pathway as well. In addition, to further confirm endothelin-1’s involvement in fibrosis, when blocking the ETA receptors with antagonists (bosentan and ambrisentan), fibroblast proliferation, α-SMA, and collagen synthesis were all inhibited, and treatment with these substances revealed restorative effects [19].

### 5.4. ET-1 in Heart Failure

Another study, which investigated the role of ET-1 in patients with heart reduced ejection fraction heart failure and the effects of dapagliflozin treatment, was performed by McMurray et al. [20]. In this study, named the DAPA-HF trial, results showed that higher baseline levels of ET-1 correlate with the severity of heart failure, as well as with a high risk of worsening, hospitalization, and death. These patients also presented with more comorbidities, worse kidney function, and lower ejection fraction. Therefore, the prognostic value of ET-1, both independently and in association with high levels of NT-proBNP (N-terminal pro-B-type natriuretic peptide) and high-sensitivity troponin-T, was also confirmed in this research [20,21].

Given its proinflammatory role, which we described above, ET-1 might also be involved in the pathophysiology of heart failure through other mechanisms. This theory could be sustained by the fact that heart failure is associated with systemic inflammation, as shown in a study published by Yuzefpolskaya et al. [22], in which the authors examined the relationship between gut microbiota and inflammation, oxidative stress, and endotoxemia in patients with heart failure. Their results show that patients with severe heart failure present higher levels of endotoxemia, inflammation, and oxidative stress, by measuring ET-1 and other biomarkers such as C-reactive protein, inteleukin-6, TNF-α, and lipopolysaccharide. Moreover, the authors determined that chronic systemic inflammation is also present following left ventricular assist device or heart transplant [22].

### 5.5. ET-1 in Oxidative Stress Induced by Cardiovascular Disease

Other than all these effects, the study by Al-kadi et al. [19] we earlier referred to in our present review, also showed that serum ET-1 levels could be tied to those of the klotho protein, an antiaging gene with antioxidant, anti-inflammatory, and antiapoptotic effects. They determined that, in cardiovascular diseases, the elevated expression of klotho protein leads to a reduced upregulation of ET-1 levels and vice versa. Therefore, treatment with endothelin system antagonists could lead to reduced oxidative stress, inflammation, and apoptosis [11].

Taking into consideration all the evidence from the referred studies, we can conclude by stating that endothelin-1 plays an important role in cardiovascular diseases. Therefore, modulation of ET-1 levels should be of great concern in this medical field [12].

## 6. Involvement in Renal Dysfunction

Given the close relationship between the vasculature and the reno-urinary system, as well as the role of endothelin-1 in vasoconstriction and inflammation, this biomarker is also involved in the pathophysiology of some kidney diseases.

Kidneys are considered the target organs of endothelins, these peptides being involved in numerous physiological and pathological processes, such as diabetic and nondiabetic chronic kidney disease (CKD), including IgA nephropathy (IgAN) and focal segmental glomerulosclerosis, as well as hepatorenal syndrome [4,18]. In the kidneys, it has been observed that, besides the contraction of the vascular smooth cells and renal blood flow regulation, ET-1 also determines the contraction of mesangial cells, regulating the glomerular filtration rate (GFR). In addition, it is also involved in fluid and electrolyte reabsorption and excretion, especially for water and sodium [4,10]. Furthermore, in patients with diabetes, treatment with ETA receptor antagonists can lead to a delay in nephropathy onset [6,20,21].

The endothelin receptors, mainly found within the kidney, are represented by ETB receptors (on the cells of the medullary collecting duct and distal convoluted tubule cells), but ETA receptors can also be found on blood vessels, podocytes, and mesangial cells. Given their location, when stimulating ETB receptors, ET-1 induces the production of nitric oxide and cGMP, leading to the inhibition of epithelial sodium channels and increased natriuresis. At the same time, stimulation of ETA receptors found inside the glomerulus can determine focal segmental sclerosis (found in models of diabetic and sickle cell nephropathy) and interstitial fibrosis. In addition, the role of ETA receptors can also be deduced by the fact that, in some studies, treatment with selective ETA receptor antagonists caused a short-term reduction in proteinuria in patients with CKD; however, their use is limited by adverse effects [18,23]. Therefore, the entire endothelin system can be involved in renal dysfunction.

As before, Table 3 presents a summary of the articles analyzed for this section of our review:

### 6.1. ET-1 in Acute Kidney Injury and Dysfunction

In the DAPA-HF trial (McMurray et al.) [20], which we mentioned earlier, the prognostic value of ET-1 was also analyzed for patients with reduced ejection fraction heart failure and kidney dysfunction. This biomarker mainly exerts its role in the proximal tubule and determines diuretic and natriuretic effects in the kidneys, which leads to reduced sodium and chloride reabsorption, lower Na+/K+ ATPase activity, and inhibits the vasopressin-induced water reabsorption in the collecting duct. It is worth noting, therefore, that given its role in the homeostasis of water and sodium, ET-1 levels are also correlated with congestion in these patients. The results showed that increased baseline values of ET-1 are also associated with a more accentuated decline in kidney function and an increased risk of hospitalization and death. However, the link between ET-1, kidney function, and heart failure is not yet fully understood [20,21].

Inside the mesangial cells, ET-1 production can also be stimulated by myoglobin. Therefore, one study by Afolabi et al. [24] investigated the involvement of ET-1 in acute kidney injury determined by rhabdomyolysis in rats. Their results show that after inducing rhabdomyolysis and in consequence, myoglobinuria, plasma levels of ET-1 are increased through an endothelin-converting enzyme 1 (ECE1)-dependent mechanism, and these levels are correlated with increased renal vascular resistance, decreased GFR, and acute kidney injury (as evidenced by determining urinary NGAL). Furthermore, in these rats, urinary levels of ET-1 and ECE1 were also increased. Moreover, by administering treatment with ECE1 and ET receptor inhibitors, kidney damage was reduced, further indicating the involvement of ET-1 in rhabdomyolysis-induced acute kidney injury. However, the mechanisms of this effect are still unknown and require further research [24].

### 6.2. ET-1 in Chronic Kidney Disease

Regarding CKD, studies show that high levels of serum ET-1 (produced by the glomerular endothelium) can lead to damage of renal podocytes, structural changes, and a decline in the barrier function and therefore, kidney function [25]. Following this premise, Hellgren et al. [25] sought to determine whether serum ET-1 levels can predict the development of this condition by measuring the levels of ET-1 in patients with CKD. Their results show that elevated values of ET-1 correlate with progression of the condition to stage 3 or higher after 10 years in women, but not in men, this association being attenuated by body mass index. Therefore, endothelin-1 could be considered a predicting factor of CKD in women [25].

Another pathophysiological mechanism, endothelin-1, is involved in kidney fibrosis in CKD, ET-1 plays a role in fibrogenesis in multiple organs and promotes myofibroblast contraction and migration [2,9,19,26]. In a study on mouse models, Arfian et al. [26] aimed to elucidate the effect of ET-1 downregulation and ECE-1 knockout on the development of fibrosis. In order to do so, they measured ECE-1 and preproET-1 mRNA expression, and they performed histopathological analysis of the kidneys in ECE-1 knock-out mice, as well as in wild-type mice after unilateral ureteral obstruction. The authors also determined ET-1 levels in the blood and kidneys in endothelin-1 knock-out mice and performed histopathological analysis after unilateral ureteral obstruction. Their results show that, in ECE-1 knock-out mice, ECE-1 and ppET-1 mRNA expression are reduced alongside kidney fibrosis, tubular injury, MCP-1 (Monocyte Chemoattractant Protein-1) mRNA expression, macrophage number, fibroblast number, and myofibroblast formation, compared to the wild-type mice. Moreover, ET-1 knockout mice had lower levels of ET-1, fibrosis, and myofibroblasts, suggesting ECE-1 and ET-1 are strongly involved in kidney fibrosis [26].

### 6.3. ET-1 in IgA Nephropathy

Other than its multiple effects described above, ET-1 can also cause podocytopathies and produce reactive oxygen species in the glomeruli. One particular form of kidney disease associated with these phenomena, in which ET-1 might also be involved, is immunoglobulin A (IgA) nephropathy [27]. In order to test this theory, Sági et al. [27] performed an analysis on ninety patients diagnosed with IgA nephropathy by measuring serum endocan, ET-1, NT-proBNP levels, and carotid-femoral pulse wave velocity, as well as performing echocardiography. After analysis, results suggested that ET-1 could serve as a biomarker to identify patients with IgA nephropathy and high risk for heart failure and/or other vascular diseases [27].

In summary, there is proof in the literature that endothelin-1 could also be involved in a multitude of pathophysiological mechanisms regarding the kidneys.

## 7. Involvement in Liver Dysfunction

During sepsis, another organ involved in its pathophysiology is the liver, with bilirubin being one of the components of the SOFA score (Sequential Organ Failure Assessment), according to the Sepsis-3 definition [28]. As ET-1 is involved in the development of sepsis, it is worth discussing whether or not it is also implicated in certain forms of liver dysfunction.

Research has shown that both endothelin-1 and its receptor subtypes are expressed in hepatocytes and hepatic stellate cells (these cells especially express ETB2 receptors, involved in vasoconstriction); however, their roles are not entirely known. ETB receptors can also be identified on liver sinusoidal endothelial cells and Kupffer cells [6,29].

One of the pathways through which ET-1 exerts its effects on the liver is by modifying the plasmatic levels of adiponectin (leading to a series of changes in systemic metabolic processes), as well as blood lipid and glucose levels via downstream effects in other tissues 6. When infused exogenously, in vitro experiments show that it concentrates inside hepatocytes and hepatic parenchymal cells, stimulating glycogenolysis and therefore increasing glucose output by activating the ETB receptors [6].

Chronic liver injury is often associated with fibrosis [30], and as we mentioned earlier in our review, ET-1 is frequently involved in the pathophysiology of fibrosis in multiple organs, including the liver. However, the mechanisms are not yet fully understood, but high levels of plasma ET-1 were identified in patients with chronic liver disease and portal hypertension [29], its levels being positively correlated with disease severity [31]. However, there is also some proof in the literature showing that circulating ET-1 levels are normal in certain cirrhosis models, such as those without hepatopulmonary syndrome (a complication of cirrhosis) [32].

In the hepatic tissue, endothelin-1 (ET-1), synthesized predominantly by liver sinusoidal endothelial cells (LSECs), acts in a paracrine manner on neighboring cell types, including hepatocytes, hepatic stellate cells (HSCs), and Kupffer cells. Kupffer cells promote ET-1 production via thromboxane A2. ET-1 contributes significantly to portal hypertension by inducing contraction of quiescent HSCs (qHSCs) and myofibroblasts [29]. At the same time, in the perisinusoidal space, HSCs act as pericytes, thus being regulated by LSEC-derived ET-1 via ETA receptors (determining secretion of fibrosis-inducing cytokines) [29,31]. Research shows that after chronic injury, hepatic stellate cells (HSCs) activate and differentiate into highly contractile myofibroblasts, becoming both a source and a target for ET-1 [29,30,31]. Furthermore, experimental data suggest that activated HSCs, but not qHSCs, are primarily responsible for ET-1 synthesis, although endotoxin (LPS) exposure can enhance ET-1 expression in both cell types [29]. HSC activation ultimately leads to cirrhosis by determining an imbalance between pro- and antifibrotic factors, a stimulated production of extracellular matrix in the perisinusoidal space, and an increased response to vasoactive mediators, like ET-1, causing structural modifications to the liver [29,31]. ET-1 can also determine contraction of the HSCs, chemotaxis, proliferation, and an increased production of collagen-I and III, as well as fibronectin [31]. ET-1-induced HSC contraction in cirrhosis is mediated by Ca^2+^-dependent MLCK activation and Ca^2+^ sensitization, involving myosin light chain phosphorylation, actin stress fiber formation, and cytoskeletal reorganization [29]. The end result is an increase in both sinusoidal and pre-sinusoidal resistance [32]. This effect further favors the development of portal hypertension [29].

In Table 4, we summarized the studies showing ET-1’s roles in liver dysfunction.

### 7.1. Involvement in Liver Fibrosis

In a study by Zhang et al. [30] on GARP-deficient mice, the authors revealed proof that, in liver fibrosis, ET-1 enhances the contractile properties of activated HSCs, while also stimulating the activation of HSCs by TGF-β [30].

Given the importance of ET-1 and ETA receptors in the activation of HSCs and progression of liver fibrosis, studies have shown that treatment with ETA receptor antagonists can ameliorate this process. In one such study, developed by ten Hove et al. [31], the authors observed that in human and murine liver fibrosis, the elevated concentrations of ET-1 and expression of ETA receptors correlated with HSC activation. The authors’ aim was to develop a novel therapeutic option involving an ETA receptor antagonist conjugated to superparamagnetic iron-oxide nanoparticles. This combination enhanced the treatment efficiency, leading to an attenuation of the fibrosis [31].

Searching for other treatment options, Lee et al. [33] also observed that auranofin, an antioxidant, could reduce the values of serum ET-1 and other fibrosis biomarkers while performing a study on mice models with nonalcoholic fatty disease. Their results indicate that this therapy suppresses ET-1 by lowering the expression of NF-κB and IkBα [33].

### 7.2. Et-1 and Liver Dysfunction in Sepsis

Regarding sepsis-associated liver dysfunction, this complication is often associated with a poor prognosis and high mortality. It may take multiple forms, such as hypoxic hepatitis, cholestasis, and/or coagulopathy. The microcirculatory failure determined by sepsis and mediated by ET-1 leads to a reduction in oxygen delivery in the liver tissue. This, in turn, determines hepatocellular injury and metabolic dysfunction [28]. According to the Sepsis-3 definition, bilirubin is the recommended biomarker for diagnosis and evaluation of this condition; a serum bilirubin value over 1.9 mg/dL determines a SOFA score of two. However, hyperbilirubinemia is a non-specific sign for this kind of dysfunction. Therefore, Woźnica-Niesobska et al. [28] developed a prospective observational study on patients with sepsis-associated liver dysfunction in order to discover novel biomarkers associated with this affliction, for early diagnosis. Given its involvement in sepsis, septic shock, and liver diseases, endothelin-1 was one of the biomarkers chosen for analysis. However, in this study, ET-1 was among the biomarkers that could not be correlated with the development of sepsis-associated liver dysfunction (only Plasminogen Activator Inhibitor 1 could predict the development of this complication) [28].

Overall, although endothelin-1 is heavily involved in fibrosis and chronic liver diseases, there is little evidence to support its implication in sepsis-associated liver dysfunction and/or other acute hepatic conditions.

## 8. Involvement in Pulmonary Dysfunction

Endothelin-1 also exerts its vasoconstrictive effects inside the lungs, affecting the entire pulmonary circulation. At this level, it determines vasoconstriction and pulmonary artery smooth muscle cell proliferation (elevated levels of ET-1 were identified in the plasma and endothelium of remodeled pulmonary microvessels of patients with pulmonary arterial hypertension, as well as chronic thromboembolic pulmonary hypertension) [34,35,36]. Furthermore, the highest concentrations of ET-1 can usually be identified inside the lungs, in both the vascular system and some structures of the respiratory system (i.e., trachea, bronchial smooth muscle) [10].

Given the fact that endothelin-1 can be found in the respiratory tract, one of its pulmonary effects is bronchoconstriction, by stimulating the contraction of the smooth muscle cells inside the airways [4,10]. However, as opposed to vasoconstriction, research shows that this effect might be mediated by ETB receptors, as treatment with ETA receptor antagonists does not exert a bronchodilatative effect [4].

Other than these effects, endothelin-1 also contributes to the regulation of airway surface secretions and fluids by stimulating chlorine secretion and modifying other epithelial ion transports in bronchial cells. This, in turn, leads to the development of fluid secretions in the respiratory tract [4,10]. Furthermore, it stimulates nasal mucus secretion and ciliary frequency of tracheal epithelial cells [10]. As a result, high levels of ET-1 were identified in bronchoalveolar lavage fluid from patients with acute asthmatic episodes and systemic sclerosis, the severity of acute asthma also correlating positively with plasmatic levels [4,19]. In addition, patients with fibrotic lung diseases and/or pulmonary arterial hypertension express higher levels of ETB receptors in the lung tissues [19].

ET-1 exerts its distinct effects in the pulmonary vasculature depending on the receptor subtype it binds to vascular smooth muscle ETA/ETB receptors mediate vasoconstriction, while endothelial ETB receptors induce NO synthesis, promoting vasodilation via the guanylate cyclase–cGMP pathway. In experimental models where common bile duct ligation was performed, increased circulating ET-1 and selective endothelial ETB upregulation (driven by hyperdynamic circulation and elevated pulmonary shear stress) led to vasodilation and hepatopulmonary syndrome. At the same time, in models with portal vein ligation, similar ETB upregulation occurs without hepatopulmonary syndrome, as circulating ET-1 levels remain normal [32].

As before, we present a summary of the articles we analyzed for this part of the review (Table 5).

### 8.1. ET-1’s Role in Pulmonary Hypertension

As mentioned above, one of the conditions frequently associated with elevated levels of ET-1 is chronic thromboembolic pulmonary hypertension. This affliction usually features microvascular damage and intimal, medial, and adventitial hyperplasia, which leads to luminal reduction. In a study performed by Feriel et al. [34] involving both human patients as well as experimental models, plasma ET-1 values were two times higher in patients suffering from the condition, in comparison to healthy subjects. Moreover, in pulmonary explants obtained from the patients, both ET-1 and ETA receptors were highly expressed. Also, ET-1 expression was elevated in human pulmonary microvascular endothelial cells when they were exposed, in vitro, to turbulent blood flow, but only in the territories affected by obstruction. Therefore, their results suggest that ET-1 does play a role in the pathophysiology of microvasculopathy and that therapy with endothelin receptor antagonists could be of use in these patients [34].

Another affliction in which ET-1 seems heavily involved, according to literature, is pulmonary arterial hypertension. This is an impairing condition, affecting the quality of life and survival. There are multiple therapeutic approaches to pulmonary hypertension, some even targeting endothelin-1 signaling (by using ET receptor antagonists mainly) [34,35,36]. Numerous studies in the literature researched the involvement of ET-1 in the pathophysiology of this condition, given the fact that this peptide is highly expressed in pulmonary arterioles of diagnosed patients, favoring inflammation and fibrosis [36,37,38,39,40]. In one such study by Maruyama et al. [37], human pulmonary arterial smooth muscle cells were stimulated with ET-1, leading to an increase in lysyl oxidase (an enzyme that catalyzes the cross-linking of collagens or elastin), which participates in the thickening of the arterial wall [37]. Moreover, the same group of researchers observed that stimulation of these cells with ET-1 also determines the activation of p38 mitogen-activated protein kinase via modified bone morphogenetic protein signaling, leading to cell proliferation inside the arteries [38].

Other studies have tried to demonstrate the role of different ET-1 genes in the pathogenesis of pulmonary hypertension and its complications. Mehra et al. [39] aimed to determine whether the development of pulmonary hypertension associated with rheumatic mitral valve disease is influenced by ET-1 and ETA gene polymorphisms in a case–control study. Their results show that, although ET-1 levels were similar in both healthy and unhealthy groups, certain gene polymorphisms (specifically recessive genotype Asn/Asn of the ET-1 gene and T/T genotype of the ETA gene) can be identified in the affected group, suggesting ET-1’s involvement in the pathophysiology [39].

### 8.2. ET-1 and Interstitial Lung Disease

Endothelin-1 has also been suggested as a biomarker in patients with interstitial lung disease, independent of the condition’s form (idiopathic pulmonary fibrosis, where ET-1 plays a profibrotic role, or interstitial lung disease associated with autoimmune diseases) [40]. In order to elucidate its involvement, Pulito-Cueto et al. [40] performed a study on a large cohort of patients with both forms of interstitial lung disease. Their results show that both groups of patients presented elevated values of serum ET-1 levels compared to healthy controls, and these values correlated with disease severity. However, the biomarker did not prove useful in order to realize the differential diagnosis between idiopathic pulmonary fibrosis and interstitial lung disease associated with autoimmune diseases [40].

### 8.3. ET-1 in Sepsis-Associated Pulmonary Dysfunction

In sepsis, as we mentioned above, the endothelium is one of the primary targets of microorganisms and inflammation. As a result of endothelial damage, plasmatic ET-1 levels are elevated, including in the pulmonary circulation. Therefore, there are multiple studies researching different therapeutic options aimed at alleviating sepsis-induced endothelial injury at this level. One such study, conducted by Lv et al. [41], sought to determine the effect of Xuebijing (a patented Chinese herbal medicine) on pulmonary endothelial damage in sepsis rat models. In their research, the treatment helped reverse the elevations of ET-1 plasma levels, suggesting that this medication could alleviate endothelial injury in early sepsis rat models [41].

### 8.4. ET-1 and COVID-19

Another condition worth mentioning, which could develop into sepsis and is associated with high levels of plasmatic ET-1, is COVID-19 [4,42]. According to proof in the literature, elevated levels of ET-1 can correlate with disease severity and mortality (alongside other biomarkers such as C-reactive protein, lactate dehydrogenase, D-dimers, sTREM-1, and hepatocyte growth factor [44,45]), research showing that healthy controls, asymptomatic patients, and those with mild symptoms present lower plasmatic ET-1 levels than those who exhibit the following:

1. Have elevated viral levels [4];
2. Require hospitalization (ET-1 could be considered an independent predictor of hospitalization) [42];
3. Develop complications [42].

Disease progression could also be determined by endothelial dysfunction [42]. These modifications can appear due to the fact that SARS-CoV-2 infection can trigger systemic inflammation, through a “cytokine storm” (possibly through the stimulated Weibel-Palade body ET-1 secretory pathway [42]), accumulation of angiotensin-II by downregulating the angiotensin converting enzyme 2, systemic thromboembolic disorders, and even pulmonary arterial hypertension, all leading to elevations of ET-1 [4]. Other theories suggest that the increased levels may be due to the release of ET-1 stored inside cells after virus-induced cell death [42]. Moreover, some studies also identified persistently elevated values of plasma ET-1 for up to 3 months post COVID-19 [43]. Some reports even suggested that treatment with antagonists of the endothelin system might have beneficial effects in COVID-19 [11].

To sum up, endothelin-1 plays a major part in a plethora of respiratory and systemic conditions that can lead to pulmonary dysfunction, being an important biomarker for endothelial injury in the pulmonary circulation and fibrosis.

## 9. Involvement in Neurological Diseases

The final system that is part of the SOFA score, by evaluating the Glasgow coma scale, is the central nervous system, systemic infections, and sepsis, which are often associated with delirium, cognitive decline, and impairment [46]. Inside the nervous system, ET-1 is expressed by endothelial cells (including in microvessels), some types of neurons, astrocytes, and epithelial cells of the choroid plexus and is involved, among others, in the function of the neurovascular unit [47,48]. When injected into the brain of rats, it determines focal ischemic lesions, and researchers are therefore obtaining stroke models [49]. In the literature, we identified multiple studies revealing neurological conditions associated with elevations of endothelin-1 in plasma and/or cerebro-spinal fluid (CSF), affecting both the central (CNS) and the peripheral nervous system (PNS), including neuroinflammation, stroke, neurodegenerative diseases, traumatic brain injury, Alzheimer’s disease, and post-COVID syndrome [47,48,49,50,51]. These studies are summarized in Table 6.

### 9.1. ET-1 in Alzheimer’s Disease

In a paper published by Asby et al. [46], they examined the neurological effects of systemic infections on patients with Alzheimer’s disease and other forms of dementia. They note that patients with Alzheimer’s disease present high values of cortical ET-1, especially in the superior temporal cortex, whether or not they associate with an infection. They also mention that these levels are more closely related to amyloid-β level and disease progression, rather than to systemic infection [46].

### 9.2. Involvement in Ischaemic Stroke

Among the top globally prevalent neurological conditions is ischaemic stroke, endothelin-1 being frequently involved in its pathophysiology [48,49,50]. Usually, astrocytes lowly express ET-1 or endothelin receptors, but these values increase in cerebral ischemia, brain injury, and encephalitis. Furthermore, ET-1 levels usually correlate with the severity of neurological deficits in patients with ischaemic stroke [48]. Considering stroke’s high mortality, researchers are actively seeking alternatives to improve therapy and prognosis. In their study, Deng et al. [48] determined that one such alternative could be Tetramethylpyrazine, a traditional Chinese medicine. By observing its effects both in vivo (on mouse stroke models) and in vitro (on cell cultures), they came to the conclusion that Tetramethylpyrazine, by lowering ET-1 expression in astrocytes, therefore inhibits ROS and oxidative stress during cerebral ischemia and attenuates blood–brain barrier damage [48].

Other than direct treatment for stroke, researchers are also seeking ways to improve present therapeutic options, such as countering the failure of reperfusion in the ischemic brain (no-reflow phenomenon) following arterial recanalization. This could happen due to microvascular changes involving ET-1 that develop during ischaemia–reperfusion. Specifically, in this case, the production of NO and peroxynitrite is highly stimulated, determining ET-1 secretion. In response, pericytes from the microvasculature express ETA receptors and contract, leading to the no-reflow phenomenon. Therefore, Yang et al. [50] performed a study on mouse stroke models to determine the effects of a drug named NA-1 when administered before recanalization. They managed to determine that this medication lowers ET-1 levels, leading to the inhibition of pericyte constriction following reperfusion, better cerebral perfusion, and decreased stroke size [50].

### 9.3. Role in Lumbar Disk Herniation and Disk Degeneration

Given its role in degenerative and neurodegenerative diseases, Uslu et al. [51] developed a cross-sectional study in order to investigate the possible link between serum ET-1 levels and the manifestations of lumbar disk herniation and intervertebral disk degeneration in human patients. In their research, they discovered that ET-1 levels were significantly elevated compared to healthy controls and that these levels correlate with the Pfirrmann grade (used to evaluate the intervertebral disk degeneration level by MRI). This suggests that ET-1 could serve as a biomarker for the degree of lumbar disk degeneration [51].

### 9.4. ET-1 in Demyelinating Disease

Another study, by Jin et al. [52], aimed to determine the involvement of ET-1 in virally induced demyelinating disease, one of the experimental models used to study multiple sclerosis in humans. In order to do so, they induced Theiler’s murine encephalomyelitis virus (TMEV) infection in mice, a condition characterized by the production of multiple chemokines in different glial cell types and activation of inflammatory immune cells, leading to demyelinating disease. The authors note that ET-1 synthesis is induced via Toll-like receptors 2, 3, and 4, while also being associated with NF-κB activation, all of which are involved in TMEV-induced demyelinating disease [2,9,33,52]. Their results show that infected mice presented higher levels of ET-1 compared to controls and that ET-1 administration accelerated clinical progression and led to an increase in cellular infiltrates inside the CNS, increased responses from inflammatory T-cells, higher levels of CCL2 and CXCL1 chemokines, IFN-γ, IL-17A, and viral RNA. Furthermore, in the group treated with ET-1, histological analysis showed elevated levels of lymphocytes and macrophages in the spinal cord and cerebellum compared to control mice, mainly in the gray matter. Moreover, therapy with endothelin receptor inhibitors ameliorated disease progression, especially when treated with the ETB receptor inhibitor [52]. This may be due to the fact that ETB are evidenced to be the predominant receptors, both in CNS and PNS [18].

### 9.5. Role in Cerebral Hemorrhage

One of the most severe neurosurgical and neurological afflictions is aneurysmal subarachnoid hemorrhage; patients often develop ischaemic complications, increasing morbidity and mortality. This condition is frequently caused by vasospasm; patients with aneurysmal subarachnoid hemorrhage and high concentrations of ET-1 in the CSF are more likely to develop vasospasm and delayed cerebral ischaemia. Therefore, Mayer et al. [53] hypothesized that treatment with an ETA receptor antagonist (Clazosentan) could prevent clinical deterioration in these patients. However, their results showed that this therapeutic option is not efficient [53].

ET-1 is also involved in the pathophysiology of hypertensive intracerebral hemorrhage. Furthermore, certain ET-1 gene polymorphisms are more closely linked to the development of this condition, as in the case of hypertension among overweight people and preeclampsia in pregnant women. Given this fact, Liu et al. [54] evidenced that the rs1920453 polymorphism in the promoter region of the ET-1 gene is significantly associated with this condition [54].

To sum up, endothelin-1 is heavily involved in the pathophysiology of multiple neurological and neurosurgical conditions, mainly through its vasoconstrictive effect in the cerebral circulation, but also plays a role in degenerative diseases. However, further research is required to analyze its full involvement in the nervous system, both in physiological and pathological conditions.

## 10. Laboratory Innovation and Emerging Methods for Plasma Endothelin-1 Quantification

In order to use endothelin-1 as a clinical tool, advances in laboratory quantification techniques need to be performed. Conventional immunoassays remain widely used but may be limited by sensitivity, specificity, and interference with other similar peptides (such as ET-2, ET-3, and their precursors), particularly when measuring low plasma concentrations of ET-1 [55]. To overcome these challenges, several innovative approaches have been reported.

One notable advance is the development of an ultra-sensitive UPLC-MS/MS (ultra-performance liquid chromatography coupled with tandem mass spectrometry) method, which enabled ET-1 quantification at picogram levels in human plasma with improved specificity compared to immunoassays, as reported by Suzuki et al. [55]. However, this technique requires costly instrumentation, skilled operators, and extensive sample preparation, which currently restricts its routine use [55].

Another innovative platform is a protein-functionalized surface plasmon resonance biosensor, according to which allows label-free and real-time detection of ET-1 binding, according to Narayan et al. [56]. This method demonstrated high sensitivity and rapid response times, with potential for miniaturization and point-of-care application. Nevertheless, the application of SPR in complex plasma samples remains technically challenging [56].

From a clinical point of view, immunoassay-based methods such as ELISA remain the most common approach. Recent studies, such as that of Dmour et al. [57], highlight the continued reliance on high-sensitivity ELISA platforms in a large patient cohort, providing valuable diagnostic and prognostic data [57]. Although less innovative than mass spectrometry or biosensor-based systems, their accessibility and practicality confer their current predominance in clinical research.

In summary, while mass spectrometry techniques offer higher sensitivity and specificity and biosensor technologies hold promise for rapid bedside application, immunoassays continue to dominate real-world use. Future developments may focus on hybrid or multiplex platforms that combine sensitivity, reproducibility, and ease of use, potentially facilitating the transition of ET-1 from research settings to routine clinical practice.

## 11. Conclusions

Endothelin-1 is one of the most potent vasoconstrictors found in the human body. Considering this effect, it is involved in multiple pathologies. In this review, we aimed to bring together the multiple effects of endothelin-1 described in the literature, more specifically in sepsis and in the systems that make up the SOFA score. Given the available research, our review could point to the idea that ET-1 might prove a valuable biomarker in the evaluation of patients with sepsis and multi-organ dysfunction. However, it is unspecific for sepsis, and we have to take into consideration the patients’ comorbidities that could interfere with its levels. Also, for the time being, there is not enough evidence in the literature to support the theory that endothelin-1 can be used as an additional criterion for determining the SOFA score. Therefore, further studies, especially meta-analyses and ROC analyses, are required in order to clearly determine this peptide’s role in septic patients.

## Figures and Tables

**Table 1:** Studies describing ET-1’s involvement in sepsis.

Authors	Study Aim	Results Regarding ET-1
Zhu, J. et al. (2022) [1]	Investigate the relationship between sepsis complicated with heart failure and the expression levels of CXCL8 ET-1 in human patients	ET-1 expression levels in sepsis patients with heart failure are significantly increased, and is an independent risk factor for heart failure in sepsis patients.
Şehitoğlu M. et al. (2023) [10]	Investigate the response after thymol treatment in sepsis model rats	ET-1 gene expression and serum levels were lower in the treatment groups and higher in the septic groups
Al-kadi, A. et al. (2025) [11]	Determine if inhibition of ET-1 signaling attenuates sepsis-induced acute cardiorenal injuries on sepsis model rats	CLP led to upregulated levels of ET-1. Treatment with ET receptor blockers improved survival, reduced the levels of inflammatory and oxidative stress parameters and improved cardiorenal functions and structure.
Xu, J. et al. (2024) [13]	Explore the link between the levels of plasma cholesterol, vascular ET-1 and the severity of sepsis in children	In the poor prognosis group, ET-1 values were significantly higher, correlating with the severity of sepsis
Lomba, L. A. et al. (2021) [14]	Evaluate the involvement of ET_A_ receptors in the febrile response induced by different doses LPS in rats	Models presented increased concentration of ET-1 in the CSF and decreased levels of big-endothelin; ET-1 is involved in the febrile response induced by LPS initially by activating ETB receptors and in later stages, through ETA receptors activation.
Macdonald, S. et al. (2022) [16]	Investigate the possible impact of IV fluids on the pathobiology of septic shock by analyzing the relationship between biomarkers of endothelial glycocalyx shedding, endothelial cell activation and IV fluid volume	Did not identify a correlation between the administered fluid volume and any of the studied biomarkers, although they did observe a significant correlation between endothelial glycocalyx shedding and ET-1.
Malheiro, L. F. G. et al. (2020) [17]	Determine if reactive hyperemia correlates with markers of endothelial dysfunction in order to identify sepsis in critical illness.	No significant differences in ET-1 levels between the control group and the septic patients’ group

**Table 2:** Studies describing ET-1’s involvement in cardiovascular diseases and cardiac dysfunction.

Authors	Study Aim	Results Regarding ET-1
Zhu, J. et al. (2022) [1]	Investigate the relationship between sepsis complicated with heart failure and the expression levels of CXCL8 and ET-1 in human patients	ET-1 expression levels in sepsis patients with heart failure are significantly increased and are an independent risk factor for heart failure in sepsis patients.
Wang, Y. et al. (2023) [7]	Evaluate the relationship between the Gensini score and ET-1 and NO serum levels in patients with chronic coronary syndrome	The high Gensini score group had higher ET-1 expressions; GS was positively correlated with ET-1
Duangrat, R. et al. (2023) [19]	Investigate the subtype specificity and signal transduction of ET receptors on fibroblast activation and myofibroblast differentiation in cardiac fibroblasts	Treatment of fibroblasts with ET-1 induced cell proliferation and synthesis of myofibroblast markers; Antagonism with ETR antagonists inhibited ET-1-induced cell proliferation and synthesis of α-SMA and collagen I
McMurray, J. J. V. et al. (2019) [20]	Evaluate the effects of SGLT2 inhibitors in patients with established heart failure and a reduced ejection fraction, regardless of the presence or absence of type 2 diabetes.	Higher baseline levels of ET-1 correlated with heart failure severity, along with a high risk of worsening, hospitalization, and death
Yuzefpolskaya, M. et al. (2020) [22]	Investigate the link between variation in gut microbiota and circulating biomarkers of endotoxemia, inflammation, and oxidative stress in patients with HF, LV assist device, and heart transplant	Severe heart failure correlates with higher levels of endotoxemia, inflammation, and oxidative stress, as shown by high levels of ET-1 and other biomarkers

**Table 3:** Studies describing ET-1’s involvement in renal diseases and renal dysfunction.

Authors	Study Aim	Results Regarding ET-1
McMurray, J. J. V. et al. (2019) [20]	Evaluate the effects of SGLT2 inhibitors in patients with established heart failure and a reduced ejection fraction, regardless of the presence or absence of type 2 diabetes	Higher baseline levels of ET-1 correlated with a more accentuated decline in kidney function and an increased risk of hospitalization and death
Afolabi, J. M. et al. (2023) [24]	Demonstrate that glycerol-induced rhabdomyolysis in Wistar rats promotes ECE-1-dependent ET-1 production, GFR decrease, and acute kidney injury	After rhabdomyolysis, plasma levels of ET-1 are increased through an endothelin-converting enzyme 1 (ECE1)-dependent mechanism; these levels correlate with increased renal vascular resistance, decreased GFR, and acute kidney injury; treatment with ECE1 and ET receptor inhibitors reduces kidney damage
Hellgren, M. I. et al. (2021) [25]	Investigate whether circulating ET-1 levels can predict chronic CKD in the population	ET-1 could be a predicting factor of CKD in women
Arfian, N. et al. (2020) [26]	Study the effect of ECE-1 knockout and ET-1 downregulation in the kidney fibrosis model in mice	In ECE-1 knock-out mice, ECE-1 and ppET-1 mRNA expression are reduced alongside kidney fibrosis and tubular injury; ET-1 knockout mice had lower levels of ET-1, fibrosis, and myofibroblasts
Sági, B. et al. (2024) [27]	Identify high-risk patients with IgA nephropathy using biomarkers, such as serum endocan, ET-1, and NT-proBNP	ET-1 could serve as a biomarker to identify patients with IgA nephropathy and high risk for heart failure and/or other vascular diseases

**Table 4:** Studies describing ET-1’s involvement in liver dysfunction.

Authors	Study Aim	Results Regarding ET-1
Woźnica-Niesobska, E. et al. (2023) [28]	Identify plasma biomarkers that could be used for an early diagnosis of sepsis-associated liver dysfunction	ET-1, the biomarkers could not be correlated with the development of sepsis-associated liver dysfunction
Zhang, X. et al. (2023) [30]	Investigate the role of Glycoprotein A repetitions predominant expressed on HSCs in the development of liver fibrosis	In mice with liver fibrosis, ET-1 enhances the contractile properties of activated HSCs and stimulates the activation of HSCs by TGF-β
ten Hove, M. et al. (2024) [31]	Investigate the therapeutic efficacy of endothelin receptor A antagonists and of ETA receptor antagonists conjugated with superparamagnetic iron-oxide nanoparticles in liver fibrosis	High concentrations of ET-1 and expression of ETA receptors correlated with activation of HSCs; the therapeutic combination led to attenuation of fibrosis
Lee, S. M. et al. (2022) [33]	Evaluate the effects of auranofin on hepatic steatosis, inflammation, and fibrosis, contributing to non-alcoholic steatohepatitis	Auranofin suppresses ET-1 and other fibrosis biomarkers

**Table 5:** Studies describing ET-1’s involvement in respiratory diseases and pulmonary dysfunction.

Authors	Study Aim	Results Regarding ET-1
Feriel, B. et al. (2024) [34]	Investigate the role of ET-1 in chronic thromboembolic pulmonary hypertension	Plasma ET-1 values were higher in patients suffering from the condition, in comparison to healthy subjects; in pulmonary explants, both ET-1 and ETA receptors were highly expressed
Maruyama, H. et al. (2020) [37]	Assess the expression of genes contributing to internal elastic lamina formation in pulmonary artery smooth muscle cells	ET-1 determines an increase in lysyl oxidase in human pulmonary arterial smooth muscle cells, leading to the thickening of the arterial wall
Maruyama, H. et al. (2022) [38]	Observe the effects of ET-1 on bone morphogenetic protein receptor expression and cell proliferation in patients with pulmonary hypertension	Stimulation of human pulmonary arterial smooth muscle cells with ET-1 determines the activation of p38 mitogen-activated protein kinase, leading to cell proliferation within the arteries
Mehra, P. et al. (2022) [39]	Study ET-1 gene polymorphisms in idiopathic pulmonary arterial hypertension associated with rheumatic valve disease	ET-1 levels were similar in both healthy and unhealthy groups, but certain gene polymorphisms can be identified in the affected group
Pulito-Cueto, V. et al. (2023) [40]	Determine the role of ET-1 as a biomarker of interstitial lung disease and its use for the differential diagnosis between idiopathic pulmonary fibrosis and interstitial lung disease associated with autoimmune diseases	Patients presented elevated values of serum ET-1 levels compared to healthy controls, and these values correlated with disease severity; did not prove useful in order to realize the differential diagnosis between the two studied conditions
Lv, J. et al. (2022) [41]	Investigate the effects of Xuebijing administration on pulmonary endothelial injury and coagulation dysfunction in the sepsis rat model	Treatment helped reverse the elevations of ET-1 plasma levels and could alleviate pulmonary endothelial injury
Abraham, G. R. et al. (2022) [42]	Determine plasma ET-1 levels in patients with COVID-19	Hospitalized patients had significantly elevated ET-1 plasma levels during the acute phase of infection
Willems, L. H. et al. (2021) [43]	Investigate endothelial dysfunction, coagulation, and inflammation long-term post-COVID-19	Elevated ET-1 levels can be identified for even up to 3 months post-COVID-19

**Table 6:** Studies describing ET-1’s involvement in neurological conditions.

Authors	Study Aim	Results Regarding ET-1
Asby, D. et al. (2021) [46]	Study the effects of systemic infection on brain cytokine levels and cerebral vascular function in patients with Alzheimer’s disease/vascular dementia	Patients with Alzheimer’s disease present high values of cortical ET-1, which are linked to disease progression
Deng, M. et al. (2025) [48]	Study the role of Tetramethylpyrazine in stroke therapy, specifically targeting ET-1 in astrocytes to alleviate ischemic brain injury	Treatment lowers ET-1 expression in astrocytes
Lui, M. et al. (2022) [49]	To induce a murine focal ischemic cortical stroke by injecting L-NAME, in combination with ET-1, into the sensorimotor cortex	When injected into the brains of rats, ET-1 determines focal ischemic lesions
Yang, X. et al. (2025) [50]	Test whether the NA-1 drug inhibits pericytic contraction of microvessels by reducing ET-1 secretion	Medication lowers ET-1 levels, leading to the inhibition of pericyte constriction following reperfusion, better cerebral perfusion, and decreased stroke size
Uslu, E. Y. et al. (2024) [51]	Research the link between serum ET-1 levels and manifestations of lumbar disk herniation and intervertebral disc degeneration	ET-1 levels were significantly elevated compared to healthy controls; these levels correlate with the Pfirrmann grade
Jin, Y. H. et al. (2020) [52]	Investigate the role of ET-1 in the development of Experimental autoimmune encephalitis and viral-induced demyelinating disease	Infected mice presented higher levels of ET-1 compared to controls, and that ET-1 administration accelerated clinical progression and led to an increase in cellular infiltrates inside the CNS
Mayer, S. A. et al. (2024) [53]	Investigate therapy with clazosentan, an endothelin receptor antagonist, in patients with aneurysmal subarachnoid hemorrhage.	Therapy is not efficient
Liu, J. et al. (2020) [54]	Examine the correlations between ET-1 gene polymorphisms and hypertensive intracerebral hemorrhage	Some ET-1 gene polymorphisms are more closely linked to the development of this condition

## Data Availability

Data sharing not applicable—no new data generated.

## Abbreviations

The following abbreviations are used in this manuscript:

APACHE II	Acute Physiology and Chronic Health Evaluation II
cGMP	Cyclic guanosine monophosphate
CKD	Chronic Kidney Disease
CLP	Cecal ligation and puncture
CNS	Central Nervous System
CSF	Cerebro-spinal fluid
ECE-1	Endothelin-converting enzyme 1
ECE-2	Endothelin-converting enzyme 2
ET-1	Endothelin-1
ET-2	Endothelin-2
ET-3	Endothelin-3
ETA	Endothelin receptor A
ETB	Endothelin receptor B
GAG	Glycosaminoglycan
GFR	Glomerular filtration rate
GPCR	G protein-coupled receptor
HSCs	Hepatic stellate cells
HUVEC	Human umbilical vein endothelial cells
IgAN	Immunoglobulin A nephropathy
IgG	Immunoglobulin G
IL-1	Interleukin-1
IL-6	Interleukin-6
IL-8	Interleukin-8
LPS	Lypopolysaccharide
LSECs	Liver sinusoidal endothelial cells
LVEF	Left Ventricular Ejection Fraction
MAPK	Mitogen-Activated Protein Kinases
MCP-1	Monocyte Chemoattractant Protein-1
MRI	Magnetic Resonance Imaging
mRNA	Messenger Ribonucleic acid
NF-κB	Nuclear Factor kappa-light-chain-enhancer of activated B cells
NO/NOX	Nitric Oxide
NT-proBNP	N-terminal pro-B-type natriuretic peptide
PNS	Peripheral Nervous System
ppET-1/preproET-1	Prepro-endothelin-1
pro-ET-1	Pro-endothelin-1
qHSCs	Quiescent hepatic stellate cells
SOFA	Sequential Organ Failure Assessment
TGF-β	Transforming Growth Factor beta
TMEV	Theiler’s murine encephalomyelitis virus
TNF-α	Tumor Necrosis Factor alpha
UPLC-MS/MS	Ultra-performance liquid chromatography coupled with tandem mass spectrometry
